# Sozialpädiatrische Versorgungssituation und -bedarfe in Zeiten der COVID-19-Pandemie 2020 bis 2022. Was wird jetzt gebraucht?

**DOI:** 10.1007/s00103-024-03847-z

**Published:** 2024-02-09

**Authors:** Elke Peters, Hannah Schmidt, Hannah Baltus, Maike Schnoor, Nina Hartmann, Alexander Katalinic

**Affiliations:** 1 Institut für Sozialmedizin und Epidemiologie, Universität zu Lübeck, Lübeck, Deutschland; 2 Klinik für Psychiatrie und Psychotherapie, Universität zu Lübeck, Lübeck, Deutschland; 3 Klinik für Kinder- und Jugendmedizin, Universität zu Lübeck, Lübeck, Deutschland; 4 Institut für Krebsepidemiologie e. V., Universität zu Lübeck, Lübeck, Deutschland; 5 Institut für Sozialmedizin und Epidemiologie, Universität zu Lübeck, Ratzeburger Allee 160, 23562 Lübeck, Deutschland

**Keywords:** Sozialpädiatrische Versorgungsbedarfe, Qualitative Studie, Experteninterview, Unterversorgte Risikogruppen, Inanspruchnahmeverhalten, Special healthcare needs, Qualitative study, Expert interview, Underserved risk groups, Utilization behavior

## Abstract

**Hintergrund:**

Kinder und Jugendliche mit sozialpädiatrischen Versorgungsbedarfen stellen eine Gruppe mit besonderen Herausforderungen dar. Ziel der qualitativen Studie war, die sozialpädiatrische Versorgung in der Pandemie aus Expert*innensicht zu beschreiben. Hieraus sollten Rückschlüsse für möglicherweise entstandene sozialpädiatrische Versorgungsbedarfe abgeleitet werden.

**Methoden:**

Es wurden 25 Expert*innen aus dem sozialpädiatrischen Bereich von Mai bis November 2022 mit leitfadengestützten Interviews zu folgenden Themen befragt: Abweichungen der Versorgung, Inanspruchnahmeverhalten von Familien, individuelle Belastungen und Ressourcen sowie nachhaltige Bedarfe. Die Interviews wurden von 2 wissenschaftlichen Mitarbeitenden inhaltsanalytisch ausgewertet.

**Ergebnisse:**

Temporär zeigte sich ein deutlich eingeschränktes sozialpädiatrisches Versorgungsangebot. Während bereits vor der Pandemie gut eingebundene Familien ausreichend mithilfe von Telefon‑/Videokontakten betreut werden konnten, wurde eine Dunkelziffer von Risikogruppen z. B. mit geringer Handlungskompetenz beschrieben, welche Leistungen nicht oder verzögert in Anspruch nahmen. Beobachtet wurden Versorgungsbedarfe für neu entwickelte psychische Auffälligkeiten und Therapierückschritte aufgrund eingeschränkter Fördermöglichkeiten sowie ein Nachholbedarf verpasster Möglichkeiten der Frühförderung bei Entwicklungsstörungen.

**Diskussion:**

Um entstandene Versorgungsbedarfe zu decken, sollten zielgerichtet unterversorgte Familien identifiziert und zeitnah unter Berücksichtigung individueller Merkmale versorgt werden. Hierzu könnten z. B. vermehrt aufsuchende Versorgungsangebote etabliert werden, die unbürokratisch bei betroffenen Familien ankommen.

## Abstract (en)

**Background:**

Children and adolescents with social pediatric care needs represent a group with special challenges. The aim of this qualitative study was to describe social pediatric care during the pandemic from the perspective of experts. From this, conclusions were to be drawn for any social pediatric care needs that may have arisen.

**Methods:**

Between May and November 2022, 25 experts from the field of social pediatrics were interviewed using guided interviews on the following topics: deviations in care, utilization behavior of families, individual burdens and resources, and sustainable needs. The interviews were analyzed by two research assistants.

**Results:**

Overall, the social pediatric care offer was temporarily clearly limited. While families who were already well connected before the pandemic could be adequately cared for with the help of telephone/video contacts, an unreported number of at-risk groups, were described, for example, those with low competence to act, who did not make use of services or did so with delays. It was observed that there was a need for care for newly developed mental disorders and regression in therapy due to limited opportunities for support, as well as a need to catch up on missed opportunities for early support in the case of developmental disorders.

**Discussion:**

To meet the needs that have arisen, underserved families should be identified and cared for promptly, taking individual characteristics into account. To this end, for example, more outreach services should be established that reach affected families unbureaucratically.

## Einleitung

Kinder und Jugendliche (KuJ) waren von (in-)direkten Auswirkungen der Coronapandemie vielfältig betroffen. Auch Eltern mussten sich veränderten Herausforderungen stellen: Neben den mit der Pandemie assoziierten eigenen Belastungen fielen auch Entlastungs- und Betreuungsoptionen weg: Reduzierte Kontakte zu Freunden und Großeltern, Schulschließungen und eingeschränkte Sport- und Freizeitmöglichkeiten führten dazu, dass die gewohnte Lebensrealität plötzlich anders bewältigt werden musste [1].

Für Familien mit KuJ, die von chronischen Erkrankungen und somit erhöhten Versorgungsbedarfen betroffen sind, stellte die Coronapandemie eine besondere Herausforderung dar. Grundsätzlich sind für diese Familien die sozialpädiatrischen Versorgungsstrukturen wichtig, die eine Vielzahl von Angeboten aufweisen. Hierzu zählen beispielsweise eine ausführliche medizinische, psychologische und soziale Diagnostik, eine bedarfsgerechte (Früh‑)Förderung der KuJ unter Einbezug von Logo‑, Physio‑, Ergo- oder Psychotherapie, eine Beratung der Eltern zu Erziehungsfragen oder sozialrechtlichen Anliegen, die Koordination und Rücksprache mit anderen Fachleuten und Einrichtungen sowie das Angebot von bedürfnisorientierten Bildungs- und Freizeitmöglichkeiten für die Familien. Diese Versorgungsstrukturen waren für einige Familien in der Pandemie temporär eingeschränkt oder wurden teilweise nicht bzw. verzögert in Anspruch genommen.

Studien deuten darauf hin, dass während der Pandemie vermehrt gesundheitliche Probleme bei chronisch kranken KuJ auftraten [2], das Risiko von Kindesmissbrauch und Gewalt gegen KuJ zunahmen [3] und sich vermehrt negative Auswirkungen auf die Entwicklung und das subjektive Wohlbefinden zeigten [4–6]. Zumindest temporär wurde ein Anstieg der Prävalenz von psychischen Belastungen bei Kindern [7] und Eltern beobachtet [8]. Betroffen waren insbesondere KuJ in Familien mit niedrigem sozialen Status, mit komplexen chronischen Erkrankungen und solche, deren Eltern psychische Belastungen zeigten [9]. In der ABCDEF-COOP-Studie zeigte sich, dass aus Sicht von Eltern der Versorgungsbedarf von chronisch kranken KuJ gegenüber nicht chronisch kranken KuJ 3‑mal so oft als ungedeckt erlebt wurde [10]. Besonders galt dies für Selbsthilfegruppen, Rehabilitationsmaßnahmen, schulische Gesundheitsdienstleistungen, Schulungen für chronische Erkrankungen sowie für psychologische Beratung/Psychotherapie [10, 11]. Zudem wurden eine geringere elterliche Lebensqualität sowie vermehrte gesundheitliche Schwierigkeiten und psychische Auffälligkeiten beim Kind berichtet [10]. Auch äußerten diese Eltern mehr Schwierigkeiten beim Zugang zu Fachärzt*innen und Notfallbehandlungen für ihr Kind [12]. Erkenntnisse über mögliche Ursachen für diese Versorgungsunterschiede liegen unzureichend vor. Auch die für Deutschland repräsentative „Studie zur Gesundheit von Kindern und Jugendlichen in Deutschland“ (KiGGS) hat keine Gründe für eine Nicht-Inanspruchnahme von medizinischen Leistungen erhoben [13]. Diskutiert werden, unabhängig von der Pandemie, soziale und sozioökonomische Unterschiede, die mit einem unterschiedlichen Inanspruchnahmeverhalten in Verbindung stehen könnten [14]. Im Zusammenhang mit der Coronapandemie können verschiedene Faktoren von Bedeutung sein: Angst vor Ansteckung, aufwendigere ärztliche Zugangsmodalitäten oder unzureichende Versorgungskapazitäten aufgrund der pandemiebedingten Maßnahmen oder einer bereits vorbestehenden Unterversorgung in bestimmten Bereichen. Insgesamt ist in der Praxis der Eindruck entstanden, dass die bereits vor der Pandemie bestehenden Herausforderungen im Versorgungssystem sich weiter zugespitzt haben, wie beispielsweise lange Wartezeiten für einen Ersttermin in einem Sozialpädiatrischen Zentrum (SPZ). Unklar ist bislang, wie die Versorgung von chronisch kranken KuJ mit ohnehin erhöhten Versorgungsbedarfen in der Coronapandemie aus Expert*innensicht im vorwiegend stationären sozialpädiatrischen Bereich verlaufen ist, welche möglicherweise ungedeckten Bedarfe entstanden sind und wie man diese decken kann.

Ziel dieser qualitativen Studie war, insbesondere die stationäre sozialpädiatrische Versorgungssituation und die nachhaltigen Bedarfe von pflegebedürftigen, schwer chronisch kranken und schwerstkranken Kindern, Jugendlichen und ihren Familien in Schleswig-Holstein in Zeiten der COVID-19-Pandemie (2020–2022) aus Sicht von Expert*innen zu erfassen. Weiterhin sollten Handlungsempfehlungen abgeleitet werden, um ungedeckte sozialpädiatrische Versorgungsbedarfe perspektivisch zu verhindern.

## Methodik

### Studiendesign.

Es wurden leitfadengestützte Expert*innen-Interviews mit verschiedenen beruflichen Expertisen durchgeführt. Der Fokus lag überwiegend auf den SPZ, da hier Kernkompetenzen der Betreuung und Koordination für KuJ vorliegen, die u. a. aufgrund von chronischen Erkrankungen ein erhöhtes Risiko für eine eingeschränkte körperliche und/oder (psycho)soziale Entwicklung aufweisen [15]. Die Leitfäden wurden auf Basis einer aktuellen Literaturrecherche partizipativ mit Mitarbeiter*innen der Klinik für Kinder- und Jugendmedizin am Universitätsklinikum Schleswig-Holstein, Campus Lübeck entwickelt.

### Rekrutierung.

Die multizentrische Rekrutierung der Expert*innen erfolgte fortlaufend von März bis November 2022 über die 4 SPZ in Schleswig-Holstein (Kiel, Lübeck, Neumünster und Pelzerhaken). Einschlusskriterien waren die Tätigkeit in einem der 4 SPZ oder in einer assoziierten Institution bzw. eine Tätigkeit als niedergelassene Pädiater*innen in Schleswig-Holstein sowie eine schriftliche Einwilligungserklärung zur Studienteilnahme. Zusätzliche Ausschlusskriterien wurden nicht definiert. Die Interviewteilnahme wurde mit 40 € honoriert. Es wurde angestrebt, möglichst heterogene Berufsgruppen zu befragen, um unterschiedliche Sichtweisen abzubilden.

### Zielgrößen.

Insgesamt wurden 4 Hauptkategorien mit zugeordneten Subkategorien als Zielgrößen in den Interviews adressiert (Tab. 1). Zusätzlich wurden personenbezogene Angaben der Expert*innen mithilfe eines Kurzfragebogens erhoben.

**Table 1:** 

*1. Sozialpädiatrische Versorgungssituation während der COVID-19-Pandemie*
Versorgungsprozesse, -abläufe und -strukturen während der COVID-19-Pandemie
Auslastung/Behandlungskapazitäten im Vergleich zur Zeit vor Corona
Kooperation/Kommunikation mit anderen Leistungsanbietern
Mehraufwand zur Aufrechterhaltung einer bedarfsgerechten Versorgung
Digitale Versorgungsangebote und deren Akzeptanz
Positive Aspekte bei sozialpädiatrischer Versorgung
Herausforderungen bei der sozialpädiatrischen Versorgung
Information über neue Coronaregelungen und Erfahrungen bei der Umsetzung
*2. Inanspruchnahmeverhalten und Zugang während der COVID-19-Pandemie*
Veränderungen im Inanspruchnahmeverhalten und Zugang
Schwierigkeiten beim Krankheitsmanagement
*3. Auswirkungen, Belastungen und Ressourcen*
Auswirkungen der Coronapandemie auf Eltern/Betreuungspersonen
Auswirkungen der Coronapandemie auf Kinder und Jugendliche
Mögliche Komorbiditäten und/oder Erkrankungen
Merkmale von Familien, die gut durch die Pandemie gekommen sind
Merkmale von Familien, die weniger gut durch die Pandemie gekommen sind
Auswirkungen der Coronapandemie und der Maßnahmen auf das Wohlbefinden der Mitarbeitenden
Coping-Strategien der Mitarbeitenden zur Bewältigung der Situation
*4. Nachhaltige Auswirkungen und Bedarfe und Wünsche*
Nachhaltig unterversorgte Risikogruppen
Unterstützungsbedarfe und Abdeckung in der Regelversorgung
Wünsche zur Deckung der Unterstützungsbedarfe
*Personenbezogene Angaben als Kontextbedingungen*
Alter, Geschlecht, Staatsangehörigkeit(en), Beruf, Berufserfahrung, berufliches Umfeld, Datum und Dauer des Interviews

### Auswertung.

Die Interviews wurden mithilfe eines digitalen Aufnahmegerätes bzw. des Videodienstleisters Webex aufgenommen und anschließend wörtlich nach Dresing und Pehl transkribiert [16]. Die Transkripte wurden von 2 Forscher*innen einer mehrschrittigen strukturierten Inhaltsanalyse in Anlehnung an Mayring [17] mit deduktiv-induktiver Kategorienbildung (Mischform) mithilfe der Datenanalysesoftware MAXQDA Plus Version 2022 (VERBI – Software. Consult. Sozialforschung. GmbH, Berlin, Deutschland) analysiert und interpretiert. Ausgewählte Ankerbeispiele (AB) illustrieren die Kategorien.

## Ergebnisse

### Studienpopulation

Insgesamt wurden 25 leitfadengestützte Expert*inneninterviews geführt, die durchschnittlich 40 min (Min.–Max.: 21–74 min) dauerten und in den Versorgungseinrichtungen (*n* = 24) bzw. per Webex (*n* = 1) durchgeführt wurden. Die Stichprobenbeschreibung ist Tab. 2 zu entnehmen.

**Table 2:** 

Merkmale der Expert*innen	*n* = 25	(100 %)
*Alter in Jahren*		
Mittelwert (SD)	48	(8,2)
Median (Min., Max.)	49	(32; 63)
*Altersgruppen*		
30–39 Jahre	5	(20 %)
40–49 Jahre	9	(36 %)
50–59 Jahre	8	(32 %)
≥ 60 Jahre	3	(12 %)
*Geschlecht*		
Weiblich	20	(80 %)
Männlich	5	(20 %)
*Nationalität*		
Deutsch	25	(100 %)
*Berufliche Qualifikation*		
Pädiater*in	11	(44 %)
Psychologe*in	5	(20 %)
Heilpädagoge*in	4	(16 %)
Ergotherapeut*in	2	(8 %)
Physiotherapeut*in	2	(8 %)
Logopäde*in	1	(4 %)
*Anzahl der Jahre im Berufsfeld*		
Mittelwert (SD)	18	(9,4)
Median (Min.; Max.)	17	(1,8; 41)
Teilnehmende Institutionen/Zuweiser*innen		
SPZ Pelzerhaken	11	(44 %)
SPZ Lübeck	4	(16 %)
SPZ Kiel	3	(12 %)
SPZ Itzehoe	2	(8 %)
Kinder- und Jugendarztpraxis (als Zuweiser)	2	(8 %)
Kinderklinik Lübeck (Diabetes-Sprechstunde)	1	(4 %)
Tagesklinik Eutin	1	(4 %)
Tagesklinik für chronisch kranke KuJ und deren Familien, Lübeck	1	(4 %)

*KuJ* Kinder und Jugendliche, *SPZ* Sozialpädiatrisches Zentrum

### Versorgungssituation während der COVID-19-Pandemie

Um die Versorgungssituation während der Pandemie zu illustrieren, sind in Tab. 3 Ankerbeispiele (AB) für alle Kategorien zu finden.

**Table 3:** 

Nr.	Ankerbeispiel (Expert*in)
1	„Manchmal kann man eben dann auch nicht wirklich gut diagnostizieren …, wenn man das Gefühl hat, ist es jetzt eine Fehldiagnose? Hätte das Kind sich vielleicht anders verhalten, wenn es meine Mimik gesehen hätte?“ (Expert*in 11; Alter 49, weiblich)
2	„Es wurde versucht, die Termine eher zu teilen, sodass nicht Arzt und Psychologe zum Beispiel an einem Termin sind, …, dass versucht wurde, schneller die Warteliste abzuarbeiten, die Briefe, die natürlich wichtig sind für die weiteren Schritte, sehr lange liegengeblieben sind, weil man sich erstmal auf die Patienten konzentriert hat, was zu vielen Problemen geführt hat in der Durchführung der Empfehlung.“ (Expert*in 27, Alter 40, weiblich)
3	„Es gibt eine Anzahl an Betten, die belegt sein müssen pro Tag, dass es reicht, um die Gehälter zu bezahlen. Wenn man darunter rutscht wegen Corona, dann kann man das einen Tag aushalten. Wenn man aber merkt, dass man pro Kind 20 anrufen muss und man hat jetzt auf einmal zehn Betten frei, die belegt sein sollten, dann weiß man, wie viele Leute man anrufen muss und dann ist auch eine Warteliste manchmal schwierig.“ (Expert*in 14, Alter 44, weiblich)
4	„Alles, was erkrankte Familie als Hilfe hatten, also sozialpädagogische Familienhilfen, Ergotherapietermine, Psychotherapie, ambulante Psychotherapie. Alles wurde – oder nicht alles, aber das meiste wurde eingestampft und auch wir in unserer täglichen Arbeit, wir sind ja so eine Schnittstelle, arbeiten nun mal auch mit dem Jugendamt, mit Schulen – es war nicht mehr möglich, mit denen zusammenzuarbeiten, weil keiner mehr kommen konnte und das Jugendamt, muss ich ganz klar sagen, war eine Katastrophe, weil sie einfach nicht mehr verfügbar waren. … So, das Einzige, was sie noch gemacht haben, waren Inobhutnahmen, was ich so mitbekommen habe, und da war man nur noch geschockt.“ (Expert*in 11; Alter 49, weiblich)
5	„… das wurde eigentlich gut akzeptiert, insbesondere da viele hier in Schleswig-Holstein sehr weit fahren, um in das SPZ zu kommen und wir teilweise Kinder von den Inseln und so weiter haben, die dann wirklich Tagesreisen unternehmen mit Übernachtung.“ (Expert*in 1, Alter 50, weiblich)
6	„Bei Diagnoseübermittlung geht die Telemedizin nach meiner Meinung gar nicht. Also ich habe jetzt gerade hier ein Gespräch gehabt, da waren wir mit mehreren Professionen dabei, weil wir eine degenerative Erkrankung der Familie mitteilen mussten, dass das kleine Kind eine Muskelerkrankung hat. Das geht gar nicht per Telemedizin, sowas geht nicht. Die brechen zusammen, die brauchen Betreuung. Denen muss man dann wirklich sofort irgendwie auch den nächsten Schritt irgendwie an die Hand geben. Das geht nicht per Telemedizin. Also sowas würde ich unverantwortlich finden.“ (Expert*in 24, Alter 60, weiblich)
7	„Ja, es ist deutlich ruhiger mit sechs Kindern als mit zwölf je nach Krankheitsbild und dann ist immer ein Riesenlärmpegel da auf Station und es war ruhig. Es war für alle gut, also auch für die Kinder. Selbst für die – man hat auch Kinder dazwischen, die ein bisschen lauter sind, aber selbst die sind ruhiger geworden.“ (Expert*in 24, Alter 60, weiblich)
8	„Also Limitationen gab es immer … warum zum Beispiel bestimmte Personen nicht in das Gebäude gelassen wurden. Manchmal war es wichtig, wenn zum Beispiel eine Heilpädagogin oder Familienhelferin, die in einer Familie ist, mit rein konnte … da gab es manchmal richtig Schwierigkeiten die mit reinzukriegen, es wurden auch oftmals Leute wieder nach Hause geschickt, manchmal kriegte man es gar nicht mit, dass da Leute nach Hause geschickt wurden.“ (Expert*in 1, Alter 50, weiblich)
9	„Es war so, dass erstmals viele Termine abgesagt wurden, was bei den Patienten oftmals Enttäuschung auch verursacht hat. Gerade die Eltern mit schwer mehrfach behinderten Kindern leben davon, auch regelmäßig angebunden zu sein. Das trägt sie sehr in ihren Sorgen und gibt Sicherheit, auch das Richtige und genügend für die Kinder zu tun. Das brach weg durch Corona und hat viele sehr hilflos gemacht.“ (Expert*in 24, Alter 60, weiblich)
10	„… In der Hochphase der Coronapandemie galten morgens andere Regeln als nachmittags und abends. Wirklich jeden Abend um 23 Uhr wurde interfamiliär bestimmt, wer jetzt bitte schön nochmal nachguckt, welche Neuigkeiten gerade erlassen wurden, die auch immer schon ab dem nächsten Tag um 9 Uhr galten, damit man sich ja nicht in eine Gefahr begab, dass man auf einmal geschlossen wird. …“ (Expert*in 14; Alter 44, weiblich)
11	„Das war aber die Kombination von ‚Es fiel erst weg‘ und ‚Sie haben dann nachher den eigenen Antrieb nicht mehr gehabt‘. Sie haben sich aber auch bei uns nicht vorgestellt innerhalb der zwei Jahre, auch Termine abgesagt und hatten Angst, sich anzustecken und so, und sind deshalb nicht gekommen.“ (Expert*in 16, Alter 45, weiblich)
12	„Also ich glaube, dass es schon bei bestimmten Erkrankungen Schwierigkeiten gab. Ich denke zum Beispiel an chronische Lungenerkrankungen wie die zystische Fibrose, dass das einfach Familien waren, wo wir alle nicht wussten, wie hoch das Risiko für diese Kinder ist, Corona zu bekommen, und wir diese eine Zeit lang zumindest hier nicht auf der Warteliste dann geführt haben für die Tagesklinik. Inwieweit es dann zu kritischen Ereignissen gekommen ist dadurch, dass sie unsere Behandlung nicht bekommen haben, das kann ich nicht sagen. Ich glaube aber, dass über die Patienten hinweg, die eben halt eine Verschiebung ihrer Behandlung erhalten haben, dass sich die Situation bei denen natürlich nicht verbessert, wenn sie nicht behandelt werden und man dann in einem halben Jahr … ein halbes Jahr für ein Kind, was 10 Jahre alt ist, eine lange Zeit ist und es eben dann halt sein kann, dass die schon in der Schule den Anschluss verpasst haben, dass sie andere Entwicklungsschritte nicht gehen konnten, und dann macht ein halbes Jahr viel aus. … Ich würde das auch bei so 20 % sozusagen sagen der chronisch Kranken. Und was man generell sagen muss, ist, dass, glaube ich, die chronisch Kranken eben, die hatten nicht nur Corona, Lockdown und so weiter, was alle anderen hatten, sondern sie haben dazu noch eine Erkrankung. Und das, würde ich sagen, ist per se einfach ein größeres Risiko, dass dadurch einfach der Schaden größer ist. … Und dass diese Patienten dann diese anderen Herausforderungen, die die Menschen haben, die keine chronische Erkrankung haben, die haben sie ja auch noch dazu. Und das, glaube ich schon, dass das das Paket einfach nochmal schwerer gemacht hat für diese jungen Leute.“ (Expert*in 18, Alter 46, männlich)
13	„Das haben die Eltern schon kommuniziert. ‚Wir können nicht mehr. Wir sind überfordert. Wir brauchen Unterstützung. Wie soll das gehen?‘ Gerade Kinder mit chronischen Erkrankungen brauchen so ihre Struktur, das gewohnte Umfeld. Brauchen ein erhöhtes Maß an Aufmerksamkeit. Das war dann schon für Eltern herausfordernd. Es sind nicht nur Schule weggefallen, sondern Logopädie, die ambulant stattfindet; Ergotherapie, die ambulant stattfinden. Es ist alles weggefallen.“ (Expert*in 22, Alter 58, weiblich)
14	„Da sind auch wirklich Situationen entstanden, wo ich mir Sorgen gemacht habe. … die Mutter eben dann dekompensiert, oder dass dem Kind wirklich was passiert. Also das war eine Mutter, die ihrem Kind bestimmt nichts Böses wollte, aber bei so einer massiven Überforderung ist es einfach – da kommen die Eltern an ihre Grenzen. …“ (Expert*in 15, Alter 48, weiblich)
15	„… die meisten unserer Kinder, da empfehlen wir oder sie haben alle einen Pflegegrad. Da war es so, dass der MDK gar nicht mehr ins Haus gekommen ist, also ein Teil haben das einfach weiter bewilligt, aber ein Teil hat einfach auch völlig willkürlich runtergestuft, was auch weniger Pflegeleistung dann für die – also greifbares Geld für die Familie bedeutet, die häufig dann nicht wussten damit umzugehen. Einen schnellen Termin jetzt hier zu bekommen tut dann Not tatsächlich. Da ist es nicht immer möglich zu priorisieren, weil die Eltern das auch gar nicht so formulieren.“ (Expert*in 13, Alter 51, weiblich)
16	„Einen geregelten Tagesrhythmus, der ist ganz, ganz wichtig. … Die Patienten aber, die vorher schon Schwierigkeiten hatten, regelmäßig die Schule zu besuchen, oder Schwierigkeiten im Sozialverhalten hatten oder in Interaktion mit anderen, oder sozial ängstlich waren, die hat es meiner Meinung nach nochmal doppelt getroffen in dieser Coronazeit, weil zum einen keine Fehlzeit in dem Sinne erfasst wurde, und dann der Druck nach Hilfe oder Behandlung sozusagen etwas rausgenommen wurde, was das Vermeidungsverhalten wieder verstärkt hat.“ (Expert*in 6, Alter 39, weiblich)
17	„Die Krankheitsbilder haben sich verschoben eindeutig, dass wird mehr – also es unterschied sich. Während den Lockdown-Phasen sind die Sozialphobiker total – denen ging es total gut, weil sie ja nicht mehr raus mussten und die haben dann nach dem Lockdown massive Probleme bekommen und während der Lockdown-Phasen sind Depressionen und so ganz schön explodiert die Zahlen der Depressionen, der Selbstschädigung und so weiter.“ (Expert*in 11, Alter 49, weiblich)
18	„… Und dann wird man einfach sehen, inwieweit das etwas ist, was mal ein Entwicklungsfenster gewesen ist, das nicht mehr zu öffnen ist, oder ob es Teile sind, die dann auch in irgendeiner Art und Weise wieder reparierbar sind. … Und die (sozial schwache Familien) werden den Schritt dann auch, selbst wenn jetzt die Normalität beginnt, nicht aufholen können. Dafür fehlt das dann an vielen Ecken und Enden.“ (Expert*in 8, Alter 55, männlich)
19	„Bei den Bewegungsstörungen ist es so, dass meistens nie irgendwas akut passiert. Da hatten sicherlich manche nicht ihre Therapien oder haben ihre Orthesen oder Hilfsmittel nicht zeitgerecht bekommen. … also es haben Verschlechterungen stattgefunden, die, wenn man jetzt aber wieder gut interveniert und ein bisschen mehr Arbeit reinsteckt … Aber es ist jetzt mit einem höheren Aufwand verbunden.“ (Expert*in 14, Alter 44, weiblich)
20	„Wenn die Familie einmal intakt und wenn sie groß ist, also wenn jetzt mehrere Generationen auf einem Hof leben. Da haben wir ganz schöne Beispiele, wo dann auch Großeltern viel übernehmen und so, und Onkels, Tanten noch dabei sind. Also wenn der Familienverbund intakt und groß ist, die hat das offenbar weniger betroffen so. …“ (Expert*in 16, Alter 45, weiblich)
21	„… schwache, einfache Familien, die finden da den Weg nicht mehr zurück, wenn sie einen verpassten Termin haben und nicht gleich, wenn sie das Sprechzimmer verlassen, einen Folgetermin in der Hand haben, den sie wahrnehmen können, dann kommen die oft nicht mehr. Das heißt, man müsste selber irgendwann nachforschen, wann wären die jetzt wieder dran. Aber bei einer so großen Patientenzahl verliert man auch als Arzt bzw. als Sekretärin, die die Einbestellungen übernimmt, verliert man den Überblick. Das hat man – das weiß man dann einfach nicht mehr. Und so sind durch Corona wie gesagt einige verloren gegangen, die sich nach einem Jahr gemeldet haben und gesagt haben ‚Mensch, jetzt waren wir schon zwei Jahre nicht mehr hier. Irgendwann möchten wir (doch) nochmal kommen.‘“ (Expert*in 23, Alter 51, weiblich)
22	„Aber eben auch dieses so ein bisschen ‚Wir gegen den Rest der Welt‘. Ich glaube, das hat auch das Team ein bisschen gestärkt, dass man gesehen hat, wie sinnig das ist, was wir hier tun“ (Expert*in 11, Alter 49, weiblich)
23	„Ja, genau. Aber das fand ich toll, dass wir gesagt haben, wir haben gemeinsam dieses Anliegen. Wir halten die Versorgung aufrecht. Wir sind da für die Eltern, für die Familien, für die Kinder und ziehen an einem Strang. Und das, fand ich, das war in dieser Zeit wirklich ganz viel wert.“ (Expert*in 20, Alter 63, weiblich)
24	„Der Anteil psychischer Erkrankungen hat drastisch zugenommen, sowohl auf Kinder- und Jugendlicher-Seite als auch auf Seite der Eltern, vor allen Dingen da bei Eltern, die vielleicht vorher schon Belastungen hatten, vielleicht auch schonmal psychisch behandelt worden waren vor zehn Jahren.“ (Expert*in 18, Alter 46, männlich)
25	„… Kollegen halt gehäuft erkrankt sind natürlich und dann länger ausgefallen sind, dass wir Kollegen hatten, die nicht mehr arbeiten durften, weil sie nicht geimpft waren.“ (Expert*in 18, Alter 46, männlich)
26	„Hm, und aber die größte Abweichung glaube ich ist eigentlich das alle noch länger auf ihre Termine warten mussten. Also hatten wir vor der Pandemie im psychologischen Bereich eine Wartezeit von neun Monaten ungefähr so sind wir jetzt bei mindestens anderthalb Jahren rein rechnerisch keine Ahnung, wo die Patienten der Warteliste noch unterkommen sollen. So, ähm, also das hat sich auf jeden Fall verändert, … dann auch schon mal die Zeiträume ein bisschen gestreckt, so dass man sie dann einfach seltener sieht und so vielleicht. Also das denke ich hat sich schon geändert einfach durch diesen ganzen Termindruck, durch ja die wenigen Ressourcen, die einfach vorhanden waren.“ (Expert*in 1, Alter 50, weiblich)
27	„… für junge Erwachsene noch ein Angebot zu machen, was am Nachmittag stattfindet, sodass … wir das noch nachholen, bevor sie uns verlassen“ (Expert*in 3, Alter 55, weiblich)
28	„Ich arbeite ganz viel im Privaten nach und bereite mich vor. Wenn man die Bereitschaft nicht hat, kann man hier eigentlich mit seinem Stundenkontingent nicht klarkommen. Ich glaube das ist ein gängiges Problem in allen SPZs.“ (Expert*in 24, Alter 60, weiblich)
29	„Also, dass wir einmal pro Schein eine Erhöhung bekämen, damit das abgedeckt wird, was wir an Leistungen machen, damit da auch eine Weiterentwicklung möglich ist. Plus, dass die Deckelung vom SPZ natürlich in Schleswig-Holstein in der Gesamtanzahl der SPZ-Scheine nicht für die Kinder ausreicht, die vorhanden sind. … es nicht damit getan ist, jetzt einfach noch irgendwelche SPZs aus dem Regen zu stampfen, für die es weder Personal noch Know-how gibt. Das würde in dem ambulanten Bereich helfen, weil man dann mehr die Möglichkeiten hätte, die Professionen noch einzustellen, die uns fehlen oder den Eltern das auch als adäquates Angebot zu machen.“ (Expert*in 14, Alter 44, weiblich)
30	„Im stationären Bereich … gibt es immer einzelne Gesetzgebungen, die für Krankenhäuser gefällt werden, aber so Spezialkliniken wie wir da überhaupt nicht darunterfallen und auch Sonderregelungen nicht zugelassen werden. Ein Beispiel, es gibt so eine eigentlich gar nicht schlechte Regelung, das Pflegegesetz, dass ab 14 Kindern eine Kinderkrankenschwester da sein muss. Klingt erstmal möglich. … Wir haben hier Rooming-In immer. Die Eltern sind da, es ist total sinnbefreit, dass – und nachts bei uns zum Beispiel jetzt die dritte Nachtwache eingeführt werden soll. … Die zieht man natürlich aus dem Tagesgeschäft raus. …“ (Expert*in 14, Alter 44, weiblich)
31	„… da sind völlig falsche Vorstellungen davon, was ein SPZ tatsächlich [braucht] …, dass man sich demensprechend auch aufstellen kann. Am Ende des Tages muss man gucken, was ist attraktiv. Warum arbeite ich wo? Wie kriege ich jemand nach … Was muss ich denen bieten? Ist das nur eine Gehaltsfrage oder ist es ein Umfeld? Ist es eine Frage letztlich der Arbeitsstruktur? … Fortbildungsveranstaltungen und so weiter. Ich glaube, da braucht man ein gutes Gesamtkonzept.“ (Expert*in 8, Alter 55, männlich)
32	„Was ich mir wünschen würde, wäre, dass man die Zeit nutzt, wenn es mal irgendwann ein Intervall gäbe, wo es keine neue Welle gibt, dass man sich darauf vorbereitet, wie man zukünftig damit umgehen möchte. Soll heißen: Ich glaube, wir sind schon weitergekommen, was die Angebote, auch die digitalen Angebote, anbelangt, aber wir sind noch lange nicht gut. Und da müssten wir, glaube ich, einfach nochmal ran, uns neue Konzepte überlegen, wie können wir unser Angebot aufrechterhalten, wenn nochmal ein Lockdown kommt und so weiter.“ (Expert*in 18, Alter 46, männlich)
33	„Die fahren vier Stunden an, dann gibt es eine Testung und dann fahren die wieder zurück. Da ist die Testung doch für – nicht verwertbar. Da muss man ein Setting schaffen, … dass die Gesundheit adäquat … versorgt wird, … dass man da unter Umständen stationäre Diagnostikaufenthalte durchführt für Patienten, die von weit herkommen. Das heißt, wenn jemand dann von den Inseln kommt, müssen die drei bis fünf Tage aufgenommen werden, damit sich die Anreise sozusagen lohnt.“ (Expert*in 8, Alter 55, männlich)
34	„Grundsätzlich würde ich mir natürlich wünschen, dass diese Sprachmedizin, die die Diabetologie darstellt und die auch einen hohen Schulungsanteil hat, sich auch abbildet in dem Personal, was man braucht, multiprofessionelles Team und Räumlichkeiten stationär wie in der ambulanten Versorgung, die das widerspiegeln. Aber so sind Kinderkliniken nicht konstruiert worden, als dass solche Räumlichkeiten da eingeplant worden sind. Eine Klinik wird zwei oder zweieinhalb Jahrzehnte vorher geplant, bevor sie fertig gebaut ist, und die Medizin von übermorgen nimmt man jetzt bei der Planung vielleicht oder hat man damals nicht so in den Fokus genommen. Aber das würde uns sehr helfen. Hätten wir einen größeren Raum, könnten wir das Schulungsdefizit schneller beheben.“ (Expert*in 3; Alter 55, weiblich)
35	„Man hat mit vielen Behörden zu tun und das ist immer anstrengend und schwierig und mühsam … dann drei Mal Widerspruch abgelehnt … für ganz selbstverständliche Hilfsmittel, weil das jemand entscheidet, der einfach überhaupt inhaltlich gar keine Ahnung hat und fachfremd ist. Das ist zermürbend, macht unsere Arbeit sehr anstrengend. … wenn es immer wieder abgelehnt wird, aber völlig klar ist, die sind auch nicht in die Familien gegangen während der Pandemiezeit. Das ist auch was, dass dann die Ärzte, die Arztberichte, die dann für solche Anträge vorliegen, immer nur aus der Warte dieses Arztes und der Thematik geschrieben sind, aber nie irgendwie alle Diagnosen übernommen werden. Das machen dann wir. Wenn sie nicht bei uns waren und unsere Berichte fehlen, kriegen sie wieder nicht den richtigen Grad der Behinderung oder Pflegegrad. Das ist was, das funktioniert wirklich nicht ganz gut.“ (Expert*in 24, Alter 60, weiblich)
36	„Jetzt ist es so, dass wenn es irgend geht, wird es telefonisch ein bisschen geklärt und ansonsten werden die Eltern mit dem Brief entlassen und müssen sich selber darum kümmern, dass es weitergeht. Also es sind schon Empfehlungen drin, natürlich, das und das und das wäre gut, aber da wir nicht mehr den direkten Austausch mit den Helfern vor Ort haben, wissen wir oft nicht, ist es überhaupt umsetzbar. Manchmal kommen auch Eltern wieder, die sagen, Ja, das ging nicht weil. So. Dann ist aber Zeit verstrichen und auch Entwicklungszeit verstrichen, also so die Möglichkeit der Weiterentwicklung für die Kinder.“ (Expert*in 15, Alter 48, weiblich)

#### Versorgungsprozesse, -abläufe und -strukturen.

Alle Expert*innen berichteten von relevanten Abweichungen gegenüber üblichen Versorgungsprozessen, -abläufen und -strukturen. Am häufigsten wurden Kürzungen und Wegfall von Versorgungsangeboten aufgrund neuer Hygienekonzepte oder unzureichend großer Räumlichkeiten benannt. Dies habe die Qualität der Versorgung deutlich eingeschränkt, da diese Angebote besonders auf soziale Teilhabe und Implementierung eines therapieadhärenten Lebensstils abzielen und wichtige Versorgungsbestandteile darstellen. Die Einbindung enger Bezugspersonen in Diagnostik und Behandlung der KuJ musste reduziert werden, u. a. durch eingeschränkte Besuchsregelungen und den Wegfall von Heimfahrten bei stationären Aufenthalten. Dies sei für die Organisation vieler Familien schwierig gewesen. Obwohl die Maskenpflicht überwiegend gut toleriert worden sei, habe diese zu Kommunikationseinschränkungen geführt. Die Umsetzung von Diagnostik‑/Interventionsangeboten (z. B. Logotherapie) sei erschwert und das Risiko für Fehldiagnosen erhöht gewesen (AB 1).

Es wurden neue (Kurz‑)Konzepte entwickelt, Arbeitsprozesse neu organisiert und Arbeitsstrukturen an pandemische Bedingungen adaptiert, um die Versorgung zu gewährleisten (AB 2).

#### Auslastung/Behandlungskapazitäten im Vergleich zu der Zeit vor der Pandemie.

Im Hinblick auf Auslastung und Behandlungskapazitäten während der Coronapandemie wurden Über‑, Unter- und Fehlbelegungen beschrieben. Drei Viertel der befragten Personen berichteten über temporäre Schließungen der jeweiligen Institutionen zu Beginn der Coronapandemie. Die Belegungskapazitäten wurden reduziert, Aufenthalte und Patientenschulungen (z. B. bei Neueinstellungen von Typ-1-Diabetes) verkürzt. Im Verlauf kam es aufgrund vieler ungeplanter Krankheitsausfälle zu finanziellen Ausfällen und Planungsunsicherheiten (AB 3).

#### Kooperation/Kommunikation mit anderen Leistungsanbietern.

Die Kooperation und Kommunikation mit anderen Leistungsanbietern wurden als enorm eingeschränkt empfunden. Insbesondere behördliche Einrichtungen seien anfangs schwierig erreichbar gewesen, sodass relevante Abstimmungen erschwert waren. Zudem seien sozialpädiatrische Leistungen eingeschränkt worden (AB 4).

#### Mehraufwand zur Aufrechterhaltung der Versorgung.

Unter dem Punkt Mehraufwand wurde von allen Expert*innen das Hygienemanagement benannt. Obwohl teilweise extra Personal angestellt wurde, wurden einzelne Mitarbeitende aus Stammteams zum Testen oder Impfen abgestellt und fehlten dadurch in der Patient*innen-Versorgung. Zudem wurde für Planung und Organisation der Einbestellung von Patient*innen aufgrund von kurzfristigen Absagen und Krankheitsausfällen ein immenser Mehraufwand betrieben. Für die Vergabe von kurzfristig zur Verfügung stehenden Terminen wurde auf bestehende lange Wartelisten zurückgegriffen. Dabei wurden Dringlichkeitsentscheidungen nach Aktenlage getroffen. Viel Zeit musste für die Suche und Gestaltung der Räumlichkeiten zur Aufrechterhaltung der Distanzierungsmaßnahmen aufgewendet werden.

#### Digitale Versorgungsangebote und deren Akzeptanz.

Dreiviertel der Expert*innen nutzten zusätzlich digitale Versorgungsangebote, wobei die Intensität und der konkrete Einsatz unterschiedlich ausgestaltet waren. Als hilfreiche Einsatzbereiche erschienen Beratungs‑, Befund- und Verlaufsgespräche sowie Fortbildungen. Die elterliche Akzeptanz wurde überwiegend als hoch eingeschätzt, da damit lange Fahrzeiten vermeidbar waren (AB 5). Als nicht hilfreich oder sogar inadäquat wurden digitale Erst- und Kennenlerngespräche sowie Diagnoseübermittlungen benannt oder der Einsatz bei unzureichend technisch versierten Eltern (AB 6). Problematisch sei die unzureichende Vergütung von Videoangeboten.

#### Positive Aspekte und Herausforderungen der sozialpädiatrischen Versorgung.

Auf struktureller Ebene wurde positiv hervorgehoben, dass Video- und Telefonkontakte die interne und externe Abstimmung erleichtern können und nach der Pandemie aufrechterhalten werden sollten. Die Pandemie habe die Digitalisierung im Versorgungsprozess beschleunigt (z. B. WLAN-Abdeckung, zusätzliche Endgeräte, Kenntnisse im Umgang mit Geräten und Software). Der Aufbau eigener frühzeitiger Impfangebote, die Verfügbarkeit von Masken, Schnelltests und gute Hygienekonzepte wurden positiv hervorgehoben. Ferner wurden die ruhigere Arbeitsatmosphäre und Möglichkeit zur sozialen Interaktion als positive Aspekte benannt (AB 7). Als herausfordernd wurde die erhöhte Bürokratie (AB 8) und der Umgang mit verunsicherten Familien beschrieben sowie die Non-Compliance von einigen Familien (AB 9).

#### Information über neue Coronaregelungen und deren Umsetzung.

Die Information über neue Coronaregeln via Newsletter und E‑Mails habe überwiegend gut funktioniert, war jedoch mit einem erheblichen Aufwand an beständiger Aktualisierung verbunden. Die Umsetzung der Regelungen habe zu erheblichen Anstrengungen geführt und war teilweise mit Sorgen vor Arbeitsstättenschließung und Konflikten unter den Mitarbeitenden verbunden (AB 10).

### Inanspruchnahmeverhalten und Zugang

#### Veränderungen im Inanspruchnahmeverhalten und im Zugang.

Das Inanspruchnahmeverhalten der Familien während der Coronapandemie wurde als heterogen erlebt. Überwiegend gelang es gut, mit Familien im Kontakt zu bleiben, die bereits fest im Versorgungssystem integriert waren. Hierzu wurde ergänzend auf Telefon- und Videokontakte zurückgegriffen. Im Gegensatz dazu verzichteten einige Familien auf stationäre Versorgung, z. B. aus Angst vor einer Coronainfektion, aufgrund von Infektionsschutzmaßnahmen (z. B. eingeschränktes Besuchs- und Heimfahrtrecht, Zutritt nur für ein Elternteil) oder bei organisatorischen Barrieren (z. B. bei eingeschränkter Geschwisterbetreuung). Dies führte zum Teil zu einer nachhaltigen Unterversorgung (AB 11).

#### Schwierigkeiten beim Krankheitsmanagement.

Alle Expert*innen berichteten, dass sie zwar keine (lebens-)bedrohlichen Schwierigkeiten hinsichtlich des Krankheitsmanagements beobachtet haben, dennoch seien die soziale Isolation und das Verpassen von sozialen Lernchancen ein entscheidender Risikofaktor für eine nachhaltig ungünstige Entwicklung der Kinder gewesen. Ein Experte berichtete zudem von mittel- bis langfristigen negativen Auswirkungen hinsichtlich des Krankheitsmanagements aufgrund von verlängerten Wartezeiten und besonderen Belastungen z. B. bei Kindern mit zystischer Fibrose (AB 12).

### Auswirkungen, Belastungen und Ressourcen

#### Auswirkungen der Coronapandemie auf Eltern/Betreuungspersonen, Kinder und Jugendliche.

Eltern wurden durch die fehlenden Betreuungs- und Entlastungsangebote, Pflege- und Fördermöglichkeiten als sehr belastet wahrgenommen (AB 13, 14, 15). Die Auswirkungen der Coronapandemie auf die KuJ mit chronischen Erkrankungen wurden als heterogen beschrieben. Insbesondere die soziale Isolation und fehlende Möglichkeiten des sozialen Lernens wurden jedoch übergreifend als große Herausforderung benannt (AB 16).

#### Mögliche Komorbiditäten und/oder Erkrankungen.

Als spezifische psychische Belastungen wurden die Zunahme bzw. Verschiebung von Krankheitsbildern sowie die Chronifizierung von depressiver- und Angstsymptomatik sowie psychosomatischen Störungen benannt (AB 17, 18). Für neuropädiatrische oder orthopädische Erkrankungen wurden Therapierückschritte in der Mobilisierung beschrieben, z. B. aufgrund von zeitverzögerten Prothesenanpassungen bei bestehenden Lieferengpässen (AB 19).

#### Merkmale von Familien, die gut bzw. weniger gut durch die Pandemie gekommen sind.

Familien, die insgesamt gut durch die Pandemie gekommen sind, zeichnen sich durch intakte familiäre Strukturen, soziale Netzwerke und Hilfesysteme, gute Handlungskompetenzen und ein hohes Selbstwirksamkeitserleben aus. Auf struktureller Ebene spielen ausreichende Wohnverhältnisse (ggf. Garten) eine bedeutsame Rolle (AB 20). Die Familien, die insgesamt weniger gut durch die Pandemie gekommen sind, zeichnen sich durch beengte Wohnverhältnisse, soziale Benachteiligung, inadäquate Unterstützungsangebote, fehlende oder unzureichende elterliche Bewältigungs‑, Handlungs- und Erziehungskompetenzen aus (AB 21).

#### Auswirkung der Coronapandemie und der Maßnahmen auf das Wohlbefinden der Mitarbeitenden und Coping-Strategien.

Die Belastungen durch die Coronapandemie wurden unterschiedlich stark wahrgenommen. Als Belastungen wurden benannt: die Konfrontation mit eigenen Ängsten und Grenzen, die Trennung von Teams sowie die damit einhergehenden Kommunikations- und Abstimmungsschwierigkeiten sowie Informationsverluste.

Als konkrete Coping-Strategien wurden insbesondere ein Sinnerleben in der Tätigkeit, ein pragmatischer Umgang mit der Situation sowie ein verbessertes Teamgefühl benannt (AB 22, 23). Weiterhin wurden die Aufrechterhaltung der Tagesstruktur sowie die Arbeitsplatzsicherheit positiv erlebt.

### Nachhaltige Auswirkungen, Bedarfe und Wünsche

#### Nachhaltig unterversorgte Risikogruppen.

Als nachhaltig unterversorgte Risikogruppen wurden am häufigsten KuJ mit psychischen Auffälligkeiten und mit psychisch vorbelasteten Eltern benannt (AB 24) sowie diejenigen, die vorpandemisch unzureichend im Versorgungsnetz eingebunden waren oder sich im Transitionsprozess befanden. Als weitere spezifische Risikogruppen mit nachhaltigem Bedarf wurden folgende Gruppen benannt: bildungsferne Familien, Alleinerziehende, Familien mit Migrationshintergrund und Pflegefamilien. Bei Familien mit Migrationshintergrund kam erschwerend hinzu, dass Dolmetscher*innen aufgrund der Distanzierungsmaßnahmen ihrer Begleitfunktion unzureichend nachkommen konnten.

#### Unterstützungsbedarfe und Abdeckung in der Regelversorgung.

Alle Expert*innen berichteten, dass der pandemiebedingte Mehrbedarf nicht ausreichend durch vorhandene Personalkapazitäten abgefangen werden konnte, zumal einige (ungeimpfte) Mitarbeitende nicht weiter beschäftigt wurden (AB 25). Die vorab schon langen Wartelisten von teilweise über einem Jahr haben sich nach Einschätzung der Expert*innen weiter verlängert. Darüber hinaus sei das Ausmaß der noch ausstehenden Unterstützungsbedarfe derzeit noch unklar (AB 26). Insbesondere für Jugendliche vor der Transition wurden Nachholbedarfe gesehen (AB 27).

#### Wünsche.

Am häufigsten wurde der Wunsch nach personeller Aufstockung geäußert, um die Arbeitslast der Mitarbeitenden und Wartezeit der Familien zu reduzieren und Nachsorgeangebote sicherzustellen (AB 28). Die Deckelung der Gesamtzahl der SPZ-Überweisungsscheine, auf deren Basis chronisch kranke und pflegebedürftige KuJ und deren Familien in den SPZ behandelt werden, müsste bedarfsgerecht aufgestockt werden (AB 29). Gleichzeitig müssten Vorgaben der Personalplanung in SPZ sich an den Bedürfnissen der KuJ und deren Eltern orientieren und nicht an den Personalvorgaben für Pflegeheime, die eine höhere Anzahl an Pflegekräften nachts erfordern. Dies wurde als wenig sinnvoll erachtet, da die Kinder in den SPZ nachts von den Eltern versorgt werden und die Fachkräfte tagsüber in der Versorgung fehlten (AB 30). Zur Anwerbung von Fachpersonal seien attraktivere Arbeitsbedingungen notwendig. Beispielsweise wurden feste Anstellungsverhältnisse, eine bessere und einheitlichere Vergütung und Fortbildungsmöglichkeiten gewünscht (AB 31).

Angeregt wird, dass die Coronapandemie auch als Chance aufgefasst werden kann, um bestehende Strukturen und Konzepte zu überdenken und zu optimieren (AB 32).

Zusätzliche Ressourcen sollten nicht nach dem Gießkannenprinzip über alle Familien verteilt werden, sondern zielgerichtet für die Versorgung von identifizierten nachhaltig unterversorgten Familien zeitnah verfügbar sein. Zudem müssten individuelle familiäre Merkmale (z. B. lange Anfahrtswege) besser berücksichtigt und aufsuchende Hilfen verstärkt werden (AB 33).

Ferner wurden Aspekte benannt, die nicht nur im Zusammenhang mit Corona stehen, jedoch einen hohen Stellenwert zur Optimierung der Versorgung aufweisen. Hierzu gehören u. a. regelmäßige Fortbildungen, ausreichende Räumlichkeiten und eine verbesserte intersektorale Zusammenarbeit über Bundeslandgrenzen hinweg (AB 34). Neben dem Abbau von Bürokratie (AB 35) wurden verbesserte Nachsorge und Rückkopplung der weiteren Versorgung gewünscht (AB 36).

## Diskussion

Das Coronavirus hat Familien mit chronisch kranken Kindern vor besondere Herausforderungen gestellt. Ziel dieser qualitativen Studie war es, insbesondere die stationäre sozialpädiatrische Versorgungssituation während der Pandemie aus Expert*innensicht darzustellen, entstandene Versorgungsbedarfe zu erfassen und Handlungsmöglichkeiten abzuleiten.

Insgesamt berichteten Expert*innen über deutliche *Abweichungen der sozialpädiatrischen Versorgung*, die insbesondere Zugangsmodalitäten, Anpassungen des Versorgungsangebots und Verkürzungen oder Ausfälle von (Gruppen‑)Angeboten betrafen. Allgemeingültige Regeln für den medizinischen und gesellschaftlichen Bereich passten häufig nicht zur sozialpädiatrischen Behandlung und mussten adaptiert werden. Ähnlich wie bei Erwachsenen mit chronischen Erkrankungen kam es zu Pandemiebeginn teilweise zur *verzögerten Inanspruchnahme* der sozialpädiatrischen Versorgung aus Sorge vor möglicher Ansteckung [18]. Auch wenn die SPZ-Versorgung zumindest bei bereits gut im Versorgungssystem eingebundenen Patient*innen weitgehend sichergestellt werden konnte, wurde die Gewährleistung eines guten Versorgungsangebots bei Erstvorstellungen und der damit verbundenen Betreuung bislang noch unbekannter Familien als herausfordernd gesehen, ähnlich wie im Erwachsenenbereich [18]. Die Ergebnisse deuten darauf hin, dass insbesondere Familien mit weniger Ressourcen *seltener Zugang zur stationären sozialpädiatrischen Versorgung *gefunden haben. Ungleichheiten in der Gesundheitsversorgung auch für Patient*innen mit medizinischen und sozialen Einschränkungen sind in der medizinischen Fachliteratur seit Jahrzehnten nachgewiesen [19]. Jeste et al. beschreiben, dass bereits im Mai 2020 36 % der Familien mit Kindern mit neurologischen Diagnosen den Zugang zu Gesundheitsdienstleistern verloren hatten [20]. Eltern von beeinträchtigten Kindern berichten in einer Studie des Fraunhofer-Instituts für Angewandte Informationstechnik (Fraunhofer FIT) und des „Inclusion Technology Labs e. V.“, Berlin, übereinstimmend mit den Expert*innen unserer Studie, dass es durch die Kontaktbeschränkungen und den Wegfall von betreuerischen Unterstützungsmaßnahmen zu einer Stagnation oder sogar einem Rückschritt in der Entwicklung der Kinder gekommen sei. Eltern selbst fühlten sich allein und überfordert [21].

Es gibt übereinstimmende Hinweise darauf, dass die COVID-19-Pandemie mit einer *erhöhten psychosozialen Belastung *für KuJ sowie deren Eltern verbunden war. Über die besonderen Auswirkungen auf Hochrisikogruppen von KuJ mit chronischen körperlichen Erkrankungen ist relativ wenig bekannt [22]. Die in den Interviews geäußerte hohe psychische Belastung der Familien deckt sich mit anderen Untersuchungen [23, 24]. Im Rahmen der repräsentativen COPSY-Studie (Corona und Psyche) zeigte sich, dass KuJ in Deutschland, unabhängig von einer chronischen Erkrankung, die COVID-19-Pandemie als mentale Belastung empfunden haben [25]. Gleichzeitig haben sich Wartezeiten auf Psychotherapieplätze weiter erhöht, die bereits vor der Pandemie sehr lang waren. Die Deutsche Psychotherapeuten Vereinigung (DPtV) bestätigte einen hohen Anteil an ungedeckten Bedarfen für kinderpsychotherapeutische Behandlungen [11]. Laut einer Mitgliederbefragung lagen die Anfragen im Juni 2022 um 48 % höher als 2020 und nur einem Drittel der KuJ konnte ein Erstgespräch angeboten werden [11]. Wie in der VOICE-Studie zur psychischen Gesundheit von medizinischem Personal während der COVID-19-Pandemie [26] berichten die interviewten Expert*innen dieser Studie von eigenen psychischen Belastungen und der Sorge vor Ansteckung von vulnerablen Personen. Neben den beschriebenen Belastungsfaktoren betonten die Expert*innen die Bedeutsamkeit eines pragmatischen Umgangs mit der Situation, Humor und Optimismus als Coping-Strategien bei der Arbeit.

Aus den Interviews lässt sich ableiten, dass Familien in der Pandemie unterschiedlich gut sozialpädiatrisch versorgt wurden und bestimmte Familien voraussichtlich längerfristig mit den *Nachwirkungen der Pandemie* beschäftigt sein werden. Das Ausmaß ist jedoch zum gegenwärtigen Zeitpunkt noch nicht absehbar. Benötigt werden vor allem Angebote, die individuelle familiäre Merkmale berücksichtigen. Hierzu müssten vermehrt Strukturen und Versorgungsangebote etabliert werden, die schnell und unbürokratisch ankommen. Zwar seien keine akuten Komplikationen aufgetreten, aber stattgefundene Verzögerungen in stationärer Diagnostik bzw. Frühförderung können sich ungünstig auf die Entwicklung auswirken. Zudem haben ungünstige Gesundheitsverhaltensweisen, wie ein sedativer Lebensstil und ungünstige Ernährungsverhaltensweisen, die bereits vor der Pandemie bestanden, weiter zugenommen. Dies deckt sich mit den Ergebnissen einer sozialpädiatrischen Beobachtungsstudie in der Adipositas-Ambulanz im SPZ der Charité – Universitätsmedizin Berlin [27]. Hier wurden erhebliche Steigerungen des Body-Mass-Indexes, eine Verfestigung ungünstiger Verhaltensweisen sowie die Zunahme des Medienkonsums während der Pandemie beobachtet [27].

Zudem war laut Expert*innen in dieser Studie eine Zunahme bzw. Chronifizierung von Verhaltensauffälligkeiten zu verzeichnen. Insbesondere für KuJ, die Zugang zum Versorgungssystem benötigen oder sich in Schwellensituationen befinden, sind zusätzlich unterstützende Angebote erforderlich. Der Übergang von vertrauten Behandelnden der Kinder- und Jugendmedizin in die Erwachsenenmedizin stellt insbesondere bei chronisch kranken Jugendlichen häufig eine herausfordernde Schwellensituation dar.

Insgesamt habe die Pandemie bereits zuvor vorhandene Probleme in der stationären sozialpädiatrischen Versorgung deutlich sichtbar gemacht. Möglicherweise führt die Verknappung von ambulanten Versorgungsangeboten und koordinierter Versorgung neben den steigenden Bedarfen zu einer Verlagerung in den stationären sozialpädiatrischen Bereich. Um nachhaltige ungünstige Auswirkungen der Pandemie im Kontext der sozialpädiatrischen Gesundheitsversorgung zu kompensieren, bedarf es zuverlässiger, langfristig tragfähiger Strukturen mit ausreichend und sinnvoll eingesetzten personellen Kapazitäten, fachlicher Expertise und vernetzten digitalen Anwendungsmöglichkeiten.

### Limitationen und Stärken

Ergebnisse aus qualitativen Stichproben sind nicht auf die Gesamtbevölkerung übertragbar. Die Interviews umfassen den Zeitraum des Ausklingens der pandemischen Lage und des Übergangs in ein endemisches Geschehen. Langfristige Folgen der veränderten Versorgungssituation und der verzögerten Inanspruchnahme von Leistungen auf die Entwicklung und das Wohlbefinden von jungen Patient*innen und deren Familien werden erst in der Zukunft sichtbar und müssen daher weiter beobachtet werden, um daraus für zukünftige Krisen oder Pandemien zu lernen. Auf eine sekundäre Quantifizierung der qualitativen Ergebnisse wurde verzichtet, da aufgrund der sehr heterogenen Berufsgruppen bestimmte thematische Aspekte per se nicht oder seltener von bestimmten Professionen benannt werden können.

Es ist gelungen, eine heterogene Stichprobe von Expert*innen aus unterschiedlichen Berufsfeldern und Institutionen (städtisch vs. ländlich), mit unterschiedlicher Berufserfahrung und unterschiedlichen inhaltlichen Schwerpunkten zu rekrutieren, sodass die sozialpädiatrische Versorgungssituation während der Pandemie breit abgebildet werden konnte. Zur Auswertung der Interviews wurde ein deduktiv-induktives Vorgehen gewählt, bei dem zur Qualitätssicherung 2 Interviewer*innen unabhängig voneinander die Auswertungen vornahmen.

## Fazit

Obwohl die sozialpädiatrische Versorgung in der COVID-19-Pandemie insgesamt ausreichend sichergestellt wurde, offenbarten sich deutliche Versorgungslücken bei bestimmten Risikogruppen. Daher bedarf es aktuell der zielgruppenspezifischen Bereitstellung von Angeboten für diese Familien zur Generierung von individuellen Hilfeleistungen. Abschließend lässt sich festhalten, dass von den Expert*innen ausreichende und sinnvoll eingesetzte personelle Kapazitäten und generell mehr Behandlungskapazitäten gewünscht werden.
